# Vitrons as Flow Units in Alkali Silicate Binary Glasses^1^

**DOI:** 10.6028/jres.065A.015

**Published:** 1961-04-01

**Authors:** Leroy W. Tilton

## Abstract

Two volume-changing processes found useful in studying the mechanism of viscous flow in glasses are: (a) stress-induced variation in fissuring near vitrons at their peripheries where the Si–O bonds are tensed and weakened, and (b) distention and modification of all silica network by added oxides. These idealized processes, readily derivable from the vitron concept of pentagonal ring structure in glass [1],^2^ are sensitive to both temperature and composition and have previously been found useful [1, 2, 3, 4, 5] for understanding other properties of glasses.

Viscous flow of vitrons as units in annealing ranges, and at higher processing temperatures for silica-rich glasses, is here viewed as permitted primarily by the breaking of tensed and weakened Si–O bonds in shell-like stressed tissues surrounding vitrons. For modifier-rich glasses at high temperatures, where the activation energy of flow is known to be low for alkali silicates but slightly higher for alkaline earths, the shearing of cation-to-oxygen bonds may also be involved. The fissures between vitrons and matrix network may provide free volume for cooperative maneuvers among the vitrons.

At high processing temperatures, both viscosity and activation energy of flow are known to decrease monotonically and at first with extreme rapidity as the content of modifiers increases. This is understandable if even slight modification materially decreases the local pliability of the matrix network and oxide-volume expansion progressively widens the fissures that must be created at vitron peripheries where the radial contraction of vitron growth potential is checked by tangential Si–O bond stresses.

On cooling toward annealing temperatures, the observed rapid increases in viscosity and in activation energy of flow are here ascribed to a general tendency toward narrowing of *peripheral* fissures, with stronger bonds therein, as the distorted dodecahedral cages return toward symmetry in response to the increases in 0–0 repulsions. Such repulsions increase with extreme rapidity if ionic separations become smaller than equilibrium distances.

Near the annealing ranges where it becomes necessary to hold glasses at constant temperatures for appreciable times in order to observe flow of the high-temperature type, it is noticeable that the activation energy of flow *increases* as the content of modifiers increases. This suggests that the closure of fissures at these very low processing temperatures may be more effective in preventing cooperative maneuvers than in resealing the weak and broken Si–O bonds.

Below annealing temperatures, where the readily observed viscosities become somewhat higher while the activation energies decrease very rapidly, it is suggested that the character of flow is changing until finally there are possible only volume relaxations with such small relative movements as are permitted by the bending rather than breaking of bonds.

## 1. Introduction

Little is known about the mechanism of viscous flow in silica-rich glasses and the random network theory has been inadequate for describing units of flow. According to the vitron concept [1] of modulated microregularity of structure which the writer [1, 2, 3, 4, 5] has applied in studies of simple silicate glasses, nearly one-third of the silica forms small and limited clusters of dodecahedral cages called vitrons (computed density 1.99 g/cm^3^ for (Si–O) = 1.60 A) within which central bonds are strong, the structure nearly regular, and the cavities large. Only oblately deformed cages can completely unite with neighbors on all sides. These microregions, the vitrons, are necessarily surrounded by relatively thin peripheral tissues or channels within which most bonds are weak and some are broken, and the structure is much less regular and partially collapsed. The vitrons and their surrounding weak tissues must be well distributed, immersed or imbedded, in what may be a matrix of “random network” in the usually accepted sense but necessarily somewhat denser than vitron structure. The thin channels and the random network are assumed to have an average density approximating 2.30 g/cm^3^ like neutron- irradiated silica as reported by Simon [6]. The random network is assumed to be stress-free with a short-range order resembling that in vitrons but with the average cavity size somewhat smaller.^3^

### 1.1. Fissuring

Vitrons as postulated are necessarily noncrystal (having five-fold symmetry) and therefore their growth is automatically limited by stresses and the peripheral bonds are necessarily weak. Consequently a corrollary of the vitron concept is a balanced system of stresses and strains involving (a) intra-tetrahedral attractive and repulsive forces tending to promote symmetry within the tetrahedra, and (b) tensed Si–O bonds maintaining the peripheral bridging contacts between tetrahedra so that the outer dodecahedral cages can exist. When temperature changes, stress-induced variations should occur in the average effective thicknesses of the thin peripheral regions where many bonds are weak and broken. In discussions of vitron theory, the occurrence of such thin regions and volume variations therein is called *fissuring.*

Fissuring may occur *laterally*, as intravitron defective regions between the outer cages of vitrons, or *peripherally* as defective links between the outer cages of vitrons and the surrounding silica network. The idea of fissuring, in general, has already been found useful in understanding the mechanical weakness of bulk specimens of silica glass and some second-order abnormal effects of temperature and pressure on its volume [1, p. 149].

Lateral intravitron fissuring is probably the chief cause of the increases (1.5 percent) in the partial molar volumes of silica found at low modifier content by Callow [7]. This lateral type is more important, volumetrically, than the peripheral type, but may have little or no effect on viscosity because lateral fissures are inclosed within the vitrons as units. In general, the peripherical fissures, which seem predominantly related to viscosity, should tend to close somewhat on cooling (whereas those of lateral type should be expected to open) under the increasing symmetrical tendencies incident to the cooling. No references to lateral fissuring will be intended in this paper unless specifically so stated.

### 1.2. Modifiers and Fissuring

According to circumstances, peripheral fissures created at high temperatures by the oblate distortions^4^ of dodecahedra necessary for vitron growth may be hampered in extent of their creation by the steric hindrance of modifiers present within the dodecahedra, and limited in their growth or maintenance by the symmetrical strength of tetrahedral units as supported by 0–0 repulsions.

Empty dodecahedra of silica glass probably deform elastically more easily than such cages with oxides therein and the dodecahedra grow together in clusters to form vitrons more easily (possibly into larger clusters). The surrounding network being also empty, its cavities likewise deform easily. Thus the distribution of stresses at vitron peripheries may be more general and greater in radial depth so that the effective peripheral shells or fissures, in which exist the critical stresses and strains, may be thicker but the individual bonds remain stronger than in modified silica glasses. Thus the viscosity of silica glass at very high temperatures becomes only moderately higher than in somewhat modified glasses at comparably high temperatures, but the activation energy remains very much higher.

In modified glasses the presence of oxides in the nonvitron cavities of the network makes the cages less pliant and deformable and thus less able to maintain good contacts with adjacent surfaces of vitrons. In other words, modified cavities of the matrix network are less adapted for closing the peripheral fissures that open as vitrons grow. This effect should be greater for oxides having large volumes or longer total extent. These views regarding the relative abilities of empty and modified cavities to influence the closure of fissures are somewhat consistent with Smyth’s^5^ mathematical analysis concerning the work done when glass is externally deformed.

According to these views, the more pliable a glass is with respect to local distortion, the greater its tendency to close peripheral fissures created by vitron growth and thus retard viscous flow of vitrons as units. Even slight modification stiffens vitron cages and matrix cavities, and especially the fissures, so that the fissures cannot so easily be closed. Hence vitron flow may be increased very rapidly and effectively by initial modification as compared with subsequent additions.

### 1.3. Temperature and Fissuring

The sensitivity of peripheral fissuring to temperature should be primarily a narrowing of fissures on cooling because of progressive return of the deformed dodecahedra and tetrahedra toward their natural symmetry as repulsions increase with extreme rapidity between the closely adjacent oxygen atoms within the tetrahedra. The narrowing of fissures should mean restoring of bond strengths. Such expectations are in general accord with the observed increase of about 50 cm^−1^ in frequency of the “valence-stretching” infrared peak near 1100 cm^−1^ upon cooling of fused silica from 1270 °C to room temperature, as reported by Neurath [8]. This contrasts with his similar finding that the cooling of two well-modified glasses from 1300 °C to room temperature causes increases of only about 25 cm^−1^ in frequency. This indicates that the fissures in well-modified glasses do not close easily and accords with the discussion above in section 1.2. on the relative properties of empty and modified cages and network cavities.

The narrowing of peripheral fissures on cooling would seem to mean increases in viscosity as well as in strength of bonds, although not necessarily so. For example, the conversion of wide gross fissures into many narrow ones, all in parallel, might eliminate critical channels for flow at low activation energy but have only moderate or small effect in increasing viscosity.

### 1.4. Oxide-Volume Expansion

As modifiers are added to silica glass there is a large structural expansion because of the added volumes that can only in part be included inside unstressed and undamaged cages of vitrons and similarly in other network cages. The mere presence of modifiers in the channels around vitrons expands these critically fissurable tissues and should be expected further to reduce Si–O bond strengths. This is in general accord with the observed decreases of about 50 cm^−1^ in frequency of the “valence-stretching” infrared peak near 1100 cm^−1^ when silica glass is very well-modified, as reported by Simon and McMahon [9] and also by Neurath [8]. There is also a structural expansion of the whole lattice or network attributable to the conventional and accepted concept of “broken bridges.” Each additional ionized oxygen means one broken Si–O bridge and two oxygens each bonded to only one rather than two silicons. This process in its initial stages is conducive to a looser and more easily distendable network. These types of gross expansion are predominently structural and together they may appropriately be termed *oxide-volume expansion* [5]. This expansion in the peripheral shells subject to fissuring is here considered as an important factor contributing to the initial precipitate decrease in viscosity and activation energy of flow as modifiers are increased.

### 1.5. Proposed Flow Mechanism

It seems very plausible that differential volume changes among diverse components of structure should affect viscosity and particularly so for a modulated regularity in structure such as is entailed by the vitron concept. The object of this paper is to see if the vitron concept and corrollary ideas readily derivable therefrom are compatible with known data on the viscosity of glasses. The data that invite analysis in this structural study are chiefly viscosities and activation energies of flow expressed as functions of temperature and of degree of modification at comparable temperatures.

Preliminary surveys of such data indicate that, in general, both viscosity and activation energy of flow increase very rapidly as temperatures are lowered in the liquid glass fields, and it has already been surmised that cooling should be expected to narrow peripheral fissures and increase viscosity and activation energy of flow. The data also indicate that at high temperatures both viscosity and activation energy of flow fall rapidly as modification is increased. In contrast, at temperatures below annealing, viscosities remain very high while activation energies of flow fall to very low values.

A remarkable point is that at high temperatures (low viscosities) the activation energy for most silicate glasses is less than half that required to break Si–0 bonds (estimated at 106 to 165 kcal/mole). Because of inability to account for this in a uniformly unstressed and strong network, Bockris and Lowe [10] and White [11] have postulated the presence in molten silica of large randomized discrete ions or “islands” and White suggests that they “permit viscous flow at lower activation energies by an ionic mechanism involving shear in the weak ionic links between anions (analogous to cleavage in crystalline silicates)”.

In accord with their postulate, but in terms of vitron theory the processes operative in the viscous flow of silicate glasses would be largely localized in, or explainable because of, such weakened connective tissues as are being suggested in this paper for uniting vitrons with a comparatively stress free matrix of a more random network. In vitreous silica these tissues may be thought of as relatively thin—an irregular series of stressed bonds from Si^+^ of one vitron to an O^−^ of a neighbor network, etc. The peripheral or “surface” bonds of the vitrons are all under very considerable tension and thus below normal strength. Small impurities of any nature, including cation oxides, could interfere seriously with these connecting links and prevent sealing of some bonds. Even such cation or oxygen (or both) as may be trapped inside the network cavities may result in fissure tissues of greater abruptness—more localized stresses and consequently fewer weaker bonds for given total strain—than for unmodified silica. Vitron cages containing oxides will have less favorable (oblate) deform ability at temperatures of formation and thus the average growth in size of vitrons may be lower as compared with conditions in unmodified and more pliable fused silica.

The flow mechanism for vitrons as units can be considered in similar manner to that sometimes given for atoms or molecules as flow units. In this case, however, many or perhaps all of the Si–O bonds near the peripheries of vitrons are already defective or very much below normal strength. Every vitron is somewhat of an island very imperfectly and perhaps somewhat loosely imbedded in a matrix of slightly denser but more pliable material.

The barriers to flow are chiefly the thin portions of the matrix network that intervene between vitrons to prevent their tangency. These portions are probably defective, little thicker than one or two tetrahedra, and they probably average between 6 and 12 vulnerable spots around the periphery of each vitron.^6^

The statistical processes continually taking place in the absence of applied stress and owing to the random energy of thermal motion, can be the making and breaking of the Si–O bonds in these vulnerable spots. The action of the externally applied stress makes it somewhat more likely for bonds to break in a direction parallel to local components of the stresses.^7^ The innumerable fissures may supply free volume to such an extent that the activation energy of flow need not be much larger than the bond energy, except in unmodified silica glass and at low temperatures.

This idea of the nature of the barriers makes it easy to understand why the activation energy of flow can vary so widely with temperature and decrease so suddenly upon the addition of very small proportions of modifiers. If one thinks of about 5 tetrahedra involved in each peripheral vulnerable spot, then it follows that all such barriers may be effectively lowered by 16.7 mole percent modification.^8^

If the vitrons are approximately alined as in the “open packing” of spheres, a shear force on one plane of vitrons pushes it over another such plane. The free volume of the innumerable fissures allows cooperative lateral movements of adjacent vitrons that minimize the cohesive forces between the hypothetical planes.^9^ For a closer packing of vitrons the required cooperative maneuvers are more extensive but of the same nature.

## 2. Viscosity Data

**Figure 1:**
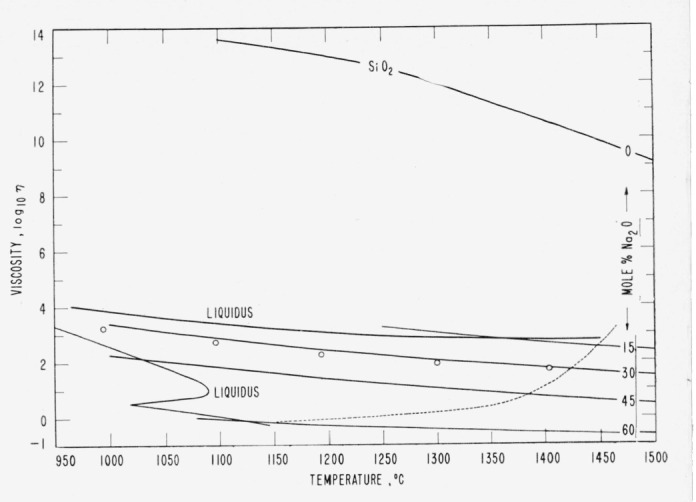
Isocomposition curves of viscosities of molten glasses in the soda silicate system. The decrease in viscosity for the addition of the first 15 mole percent Na_2_O is extremely large compared to subsequent equal additions. Curves for 15, 30, 45, and 60 mole percent Na_2_O are taken from data by Heidkamp and Endell [12]; circles show data by Shartsis, Spinner, and Capps [31] for 30.1 mole percent Na_2_0; the SiO_2_ curve is from Solomin [13].

Measurements of viscosity of silicate glasses have been made over a range from a fraction of 1 poise to 1×10^20^ poises.^10^ This remarkably wide range in this property is often overlooked because we usually plot viscosity on a logarithmic scale, or use only the logarithm of viscosity. The rapid *thermal* decrease in viscosity with increase in temperature, especially for molten glasses, is well known, but there is also an extremely rapid structural decrease in viscosity as modifier oxides are added to silica glass and particularly so for the initial additions. This is shown in figure 1 where isocomposition curves for 0, 15, 30, and 60 mole percent Na_2_O are plotted from the data of Heidtkamp and End ell [12] and of Solomin [13].

### 2.1. Temperature Effects

The concomitant effects of temperature should be eliminated before plotting viscosity as a function of composition in order to study possible effects of Assuring and oxide-volume expansion. It is evident that temperature effects are not removed satisfactorily for comparisons of different glasses merely by using a constant temperature. It is sometimes suggested that equality of *dη/dt* is a reasonable basis for the selection of “corresponding temperatures” where *η* represents viscosity and *t* is temperature. Such a criterion may be satisfactory for a comparison of structurally similar glasses. But whether one uses a constant temperature (ordinates in fig. 1) or temperatures of equal slope (see for example, dashed line in fig. 1), it seems that the very great differences in viscosity between silica glass and one containing only a few percent of Na_2_0 must be chiefly of structural rather than thermal origin.

Another possibility for comparing the viscosities of compositionally different glasses involves use of the exponential equation

η=Aexp(B/RT) (1)

which has been much used for viscosities, also for ionic conductivities and diffusion phenomena in glasses. In this equation, *A* is a constant relating to frequency of the reaction, *B* is the activation energy for the process (*R* being the gas constant), and *T* is absolute temperature. This equation has been found valid for viscosities of nonpolymerized liquids over wide ranges of temperature.

**Figure 2:**
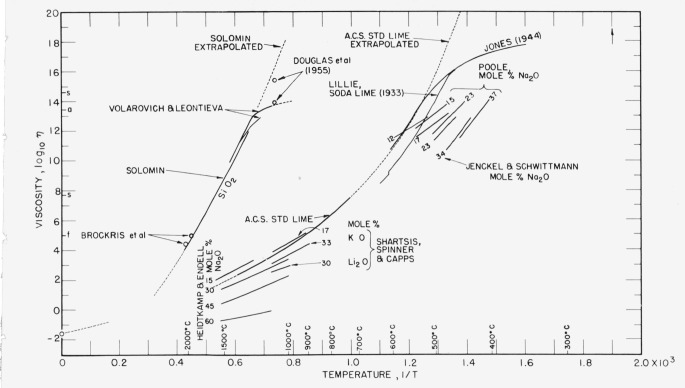
Effect of modifiers on viscosities of silicate glasses. Note that modifiers reduce temperatures for isoviscous flow; that curves converge toward points at very low viscosities and high temperatures; also toward points at’very high viscosities and low temperatures. The best formula for representing the viscosities of the American Ceramic Society’s standard lime glass in fluid condition extrapolates to log *η* = − 1.6 at infinite temperature, and to infinite viscosity at 250 °C. The same form of equation was used by Solomin [13] for SiO_2_. (For data see literature ref [12, 13, 18 19, 24, 25, 26, 31, 33, 36].)

For glasses this equation has been much used over short temperature intervals, especially at and near annealing regions where viscosities range from 1×10^15^ to 1×10^13^ poises. It is used also at the much higher molten-glass temperatures, but different constants are necessary in these two temperature regions. This will be evident from figure 2 if it be remembered that the straight-line equation

logη=logA+(B/RT) (2)

which relates viscosity and reciprocal of absolute temperature is equivalent to eq (1), and consequently *d* log *η/d*(1/*T*) = *B/R*. Figure 2 emphasizes the great difference between viscosities of molten glasses and so-called viscosities of glasses below their annealing ranges.

Several investigators, including Douglas [14], Cox [15], and Hodgdon and Stuart [16] have discussed equations for relating viscosity and temperature, but, as pointed out by Anderson and Stuart [17], these equations in each case reduce to 4-constant equations comparable with a dual writing of eq (1), once for high temperatures and again for low temperatures.

Others including Lillie [18] have sought to regard the constant, *A*, as a function of temperature, and Jones [19b] has suggested a continuous distribution of activation energies. Plumat [20] finds evidence of a step-wise distribution and “critical temperatures.” An important clue for clarification may be recognition of the dominant role of the volume in determining the internal mobility of simple nonpolar liquids. One might follow Fox and Flory [21] in supposing that viscosity changes with temperature through two distinct effects: (1) the thermal energy available, and (2) the contribution of free volume to height of barrier. According to this idea one might seek to understand the inflections on some curves such as those of figure 2 at or near lower annealing 11 temperatures (where log *η* = 13 approximately) if during cooling the bond-angle (3,4,5) configurational decreases in volume^11^ are rapidly checked and an expansion by lateral fissuring between cages of vitrons is increased because of enforced quasi-rigidity and the stress-symmetry relationships.

Possibly all such views are more nearly consistent than their formal presentations indicate. However, there seems little assurance that the use of eq (1) can be entirely satisfactory for eliminating all temperature effects in the general comparison of glasses having different compositions.

**Figure 3:**
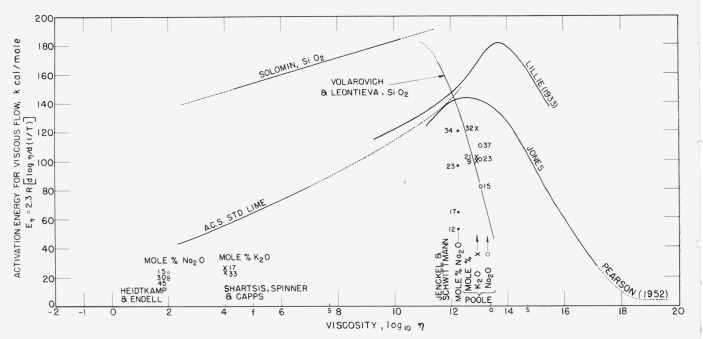
General effect of modifiers on activation energy for viscous flow in molten and quasi-rigid silicate glasses. At temperatures where glasses are molten the activation energies are decreased by addition of modifiers but at lower temperatures the energies are raised by addition of modifiers. It is suggested that the high-temperature process of fissuring at the peripheries of vitrons is reversed on cooling by restored symmetry that is progressively enforced by the 0–0 repulsions, especially on near approach to quasi-rigid conditions. (For data see literature ref [12, 13, 18, 19, 24, 25, 26, 31, 34].)

Still another expedient for a survey of structural relationships, perhaps at approximately comparable temperatures, is exhibited in figure 3 where a number of computed values of activation energies have been plotted against log *η* to emphasize or accentuate the differences in flow properties above and below those regions where glasses change from predominantly fluid to quasi-rigid materials. That maximum values of the activation energy of flow occur in or near the annealing range (log *η* = 13 in fig. 3) has been explicitly noticed by Jones [19a] and by Stanworth [23c]. The maxima, of course, correspond with the inflections that are prominent in figure 2. The decrease to very low values at much lower temperatures, while viscosity remains high, suggests that the type of flow, if any, that occurs at very low temperatures is probably very different from that at high temperatures.

**Figure 4:**
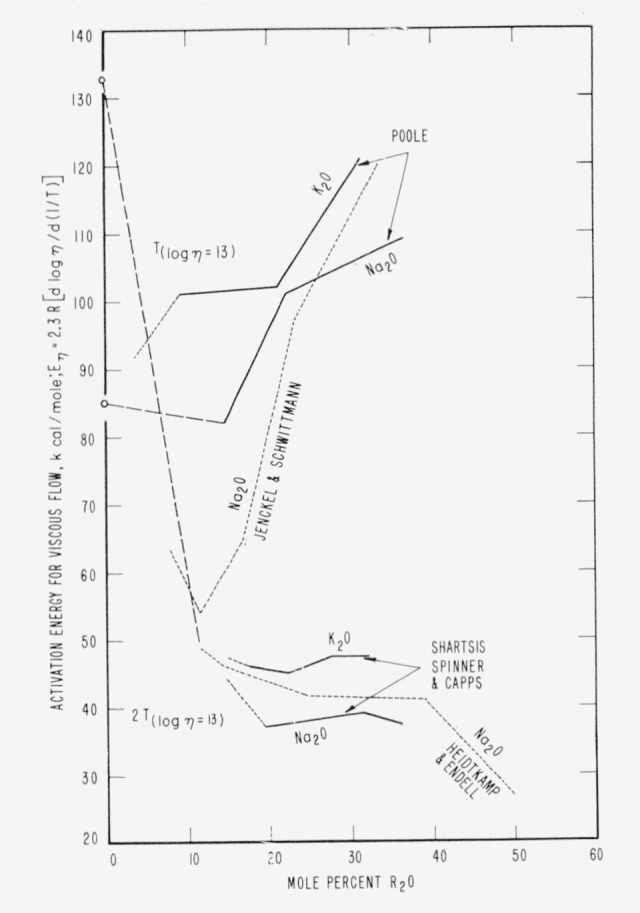
Activation energies for isoviscous flow near annealing temperatures contrasted with those at twice annealing temperatures (absolute scale). For quasi-rigid glasses note the rising trends. For fluids there is a very sudden drop until cages are approximately filled with oxides; then a near steady state until cages are completely saturated. This confirms the reversal in structural effects noted in figure 3 and text. (For data see literature ref [12, 24, 25, 31].)

In figure 3 a number of points are plotted for several degrees of modification of various glasses, some for high temperatures and some for annealing temperatures. It will be noticed that higher oxide content correlates with lower activation energy of flow in liquid silicates but with higher activation energy in quasi-rigid glasses very near the annealing temperatures.^12^ Similar data have been plotted in figure 4 to confirm this constrast between annealing and much higher temperature.

This reversal in structural effect seems, to a first approximation, to occur regardless of the type of modifier. At the lower temperatures it is especially difficult to explain the higher activation energy with higher oxide content on the basis of a continuous random network because modifiers are supposed to disrupt and weaken the structure as compared with that in pure fused silica. On a vitron basis, however, the processes of fissuring and oxide-volume expansion can serve to explain the reversal if it be remembered that the flow of vitrons requires not only that bonds between vitrons and matrix be broken but also that free volume be made available for the necessary cooperative maneuvers. At high temperatures the modifiers by their mere presence can cause extra tension and weaker bonds in critical fissures while free volume can be available by widely extended cooperative maneuvers. At low temperatures the modifiers are trapped in the fissures while they are being closed by the 0–0 repulsion effect and Si–O bond-angle contractions—a closure that may be much more effective in preventing cooperative maneuvers than in resealing weak and broken bonds. Thus the *E_η_* curves for different modifications could cross and the correlation between *E_η_* and degree of modification be reversed from decidedly negative at very high temperatures to decidedly positive at and near annealing.

Such a reversal effect, if any, in respect to log *η* is not apparent from available data. Indeed, its existence for *E_η_* may be partly an evidence of a change from high-temperature to low-temperature types of flow in silica-rich glasses that takes place gradually at temperatures between the softening and strain regions where values of *E_η_* have their maxima.

This analysis of viscosity data, as presented in figures 2 and 3, suggests that there are both similarities and well-marked differences in the phenomena that have been designated as viscous, especially near annealing temperatures, in silicate glasses. Here it seems pertinent to recall that Lillie [18] prefers the equation

$$\log\;\eta =A+B/\left(T-T_{0}\right)$$ (3)

for viscosities between 10 and 10^12^ poises, and says it is the only relationship between viscosity and temperature that fits high temperature data “within the expected limits of experimental error.”

Although Lillie transforms eq (3) so that *A* becomes a function of temperature, the equation can be written also as

$$\eta =A\prime \exp\left(\frac{B\;T}{T-T_{0}}\cdot \frac{1}{T}\right)$$

showing that the activation energy can be considered to increase as temperature is lowered. The values of *T*_0_ required for this equation are well below temperatures in the annealing ranges. Possibly these *T*_0_ temperatures can be regarded as ultra lower limits for viscous flow of a high-temperature type in which bonds at vitron peripheries are broken; limits where activation energy for such flow might become extremely high (because of position barriers) provided requisite, impossibly long periods could be taken for observations. Actually, Isard and Douglas [36] have reported for fused silica at 1080 °C both a low-temperature type of (high) viscosity immediately observed, and a high-temperature type of (even higher) viscosity observed after a holding time of 30 hours. Both points are shown in figure 2, the first agreeing with an extrapolation of results by Volarovich and Leontieva, the other with an extrapolation by eq (3) as used by Solomin with *T*_0_ = 455 °K.

In contrast with the high and increasing activation energies of flow at equilibrium, according to eq (3), the activation energies of “flow” immediately observable in the really low-temperature region decrease and become of the order of magnitude for bending or stretching rather than breaking of bonds, and the number of such bonds that bend or stretch probably becomes extremely large. As mentioned above, this low-temperature flow could be the volume relaxation connected with the closure of peripheral fissures and the opening of lateral fissures consequent on the enforced further symmetry as cooling progresses. Such relaxation^13^ during cooling has previously been mentioned by the writer [1, p. 149] and its minor contractive aspect through (peripheral) fissuring associated with the decrease in activation energy of flow near annealing temperatures. Also, its major expansive aspect through (lateral) fissuring was suggested [1, p. 150] as chiefly responsible for the very small and abnormal negative coefficient of equilibrium expansion as observed by Douglas and Isard [37] between 1000 ° and 1500 °C.

### 2.2. Structure Effects

The isotherms of activation energy of flow as plotted against mole percentage of various oxide modifiers in series of binary glasses have received only moderate attention. Stanworth [23a] gives activation energy data on a few glasses by Taylor and Dear [27] and by Taylor and Doran [28] for the transformation range, and MacKenzie [29] gives data for, or near, 1400 °C. Stanworth [23b] also reproduces such a curve published by Preston and Seddon [30] based on the viscosity measurements by Heidtkamp and Endell [12] at 1000 to 1500 °C. The Preston and Seddon energy curves agree more or less with MacKenzie’s curve. Other data by Shartsis, Spinner, and Capps [31] are used by White [11] in similar curves that include the MacKenzie and other data.

**Figure 5:**
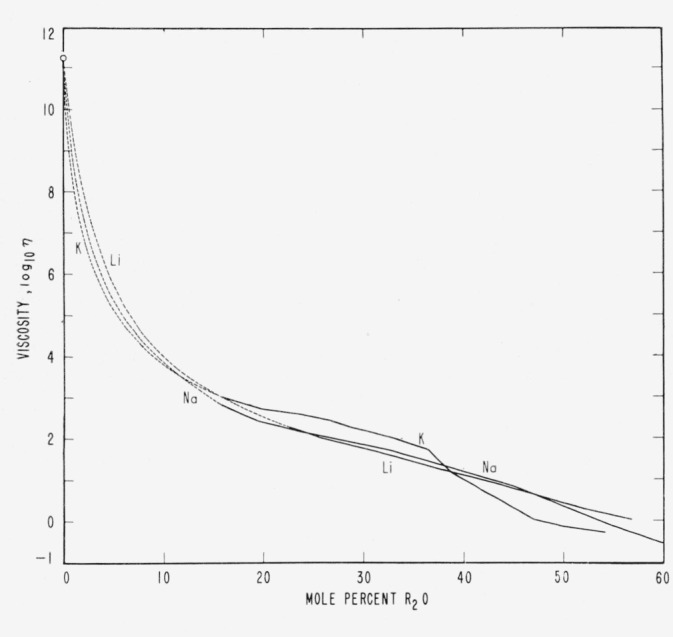
Differential effects of modifier oxides on isothermal viscous flow of molten glasses. (Averages of data by Endell and Hellbrügge [35] at 1,250 to 1,450 °C.) Even slight oxide-volume expansion in the imperfect and denser network surrounding vitrons may localize and expand the peripheral fissures created by vitron growth. The largest K_2_O oxide is most effective. The rapid decrease in viscosity is checked before 16.7 mole percent R_2_O where one oxide molecule is available for every 5 of SiO_2_. Thereafter all cages can offer some resistance to the distortion necessary for vitron growth and the formation of wider peripheral fissures.

**Figure 6:**
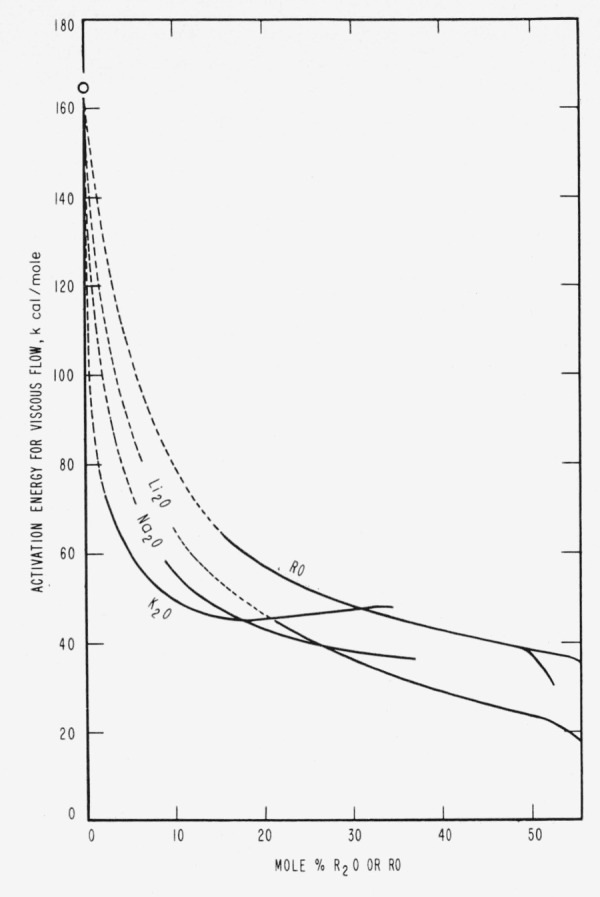
Differential effects of modifier oxides on activation energies for isothermal viscous flow of molten glasses (1,400 °C). (From White’s thesis [11].) Note the sudden drop increasing in the order Li, Na, K of increase in size of the oxides. After oxide saturation the descent is much less rapid, perhaps because fissures remain relatively constant and the activation energy is chiefly that for the breaking of cation-to-oxygen bonds.

From figure 5 and from figure 6 (which is reproduced from figure 5 of White’s [11] thesis), it is noticeable that the respective curves for K, Na, and Li complete their rapid descent and cross each other in the region near 16.7 mole percent where one oxide molecule is available for each dodecahedral cage, or every 5 SiO_2_.^14^ Then it seems from the curves for the activation energy of flow (which descend only a little further) that the glass structure remains relatively constant until further addition of modifiers up to at least 28.6, 37.5, and 50.0 mole percentages of K_2_0, Na_2_0, and Li_2_0, respectively, which are the effective saturation limits for cation in the cages as previously deduced by the writer [3] from the data of Preston and Turner [32] on 20 hour volatilization losses at temperatures from 1100 to 1400 °C.

The marked changes in slope of the curves of figures 5 and 6 may indicate that two structural processes or factors are operating to advance viscous flow at high temperatures. One predominates in the high silica glasses with low modifier content, and the other process predominates after modification progresses beyond 16.7 mole percent.

The first factor, which acts very precipitately, may be fissuring combined with oxide-volume expansion in the fissures. This process, connected with or evidenced by structural changes in volume, predominates where the activation energies are sufficiently high to include the breaking of some Si–O bonds, especially those not of full strength because of (1) fissuring and (2) fissures enlarged by oxide-volume expansion. In this low-modifier composition range, the viscosity and the activation energy for viscous flow fall more and more rapidly in the order Li, Na, and K (fig. 5), corresponding to the increasing volumes of the oxides that could be trapped in the fissures to prevent their normal closure, or trapped in the nonvitron cavities to make the network less pliable locally and less conducive to fissure closure.

At a concentration of 16.7 mole percent modifier, there will be one oxide molecule for every 5 tetrahedra. All cavities should be less deformable; the vitrons might cease growing at smaller diameters; the fissures should be wider; and the viscosity therefore could be much lower than for unmodified glass. From figure 5 (see also fig. 1) it can be estimated that the initial rapid structural-change process is essentially completed. The decreases of viscosity and of activation energy of viscous flow in the higher modifier range of composition are very gradual and may be caused by broken oxygen bridges and by steric hindrance of many modifiers trapped in the fissures.

In figure 6 it seems that at all degrees of modification the activation energy of flow falls appreciably slower for the alkaline earth oxides, which have smaller molar volumes and smaller maximum dimensions than any of the alkali oxides. This agrees with expectations that in silica-rich glasses the smaller sizes and volumes would make the earths less effective in promoting and maintaining fissuring. In the well-modified glasses (where flow may be effected by the shearing of weakened cation-to-oxygen bonds), it should be expected that divalent cations, each bonded to two unshared oxygens, can offer somewhat greater barriers to flow than the monovalent cations.

These structural interpretations accord with and tend to clarify the meaning implied by Shartsis, Spinner, and Capps [31] when they showed conclusively that “to a first approximation viscosity in these [alkali silicate] systems is a function of the number of alkali ions *per unit volume* and is almost independent of the type of alkali ion.” (The effect of type of ion could be implicit, however, in that it can affect the volume.)

In contrast with these interpretations based on vitron theory, the most widely offered explanation of the decreases in viscosity upon the addition of modifier oxides is the concept of broken oxygen bridges. At high modifier contents, especially near 67 mole percent nonsilica where all 4 corner bridges of the tetrahedra are broken and the viscosity is less than one poise, this view is tenable, but the case seems very different for silica-rich glasses. At 16.7 mole percent modifier, where only 10 percent of all oxygen bridges can possibly be broken (even according to conventionally accepted estimates^15^), the viscosity at 1200 °C has decreased from 10^13^ poises for pure silica to 10^3^ poises for a melt of soda silicate glass (fig. 1). The decrease is extremely precipitate for the first few percent of added oxide 16 and relatively very slow after 16.7 percent (fig. 5).

Certain data from infrared studies may be cited here to illustrate the possible bearing of such investigations on structural conditions in glasses. From figure 6 (1400 ° C), the decrease in activation energy of flow at 16.7 percent modification is roughly 90 percent of the total observed decrease for full or maximum modification, and it has here been assumed that the energy of activation is that for the breaking of stressed Si–O bonds. In contrast, for room temperatures it has been shown by Simon and McMahon [9] that the decrease in wave number of the “valence-stretching” peak in the infrared near 1100 cm^−1^ is, for 16.7 percent modification, only about 30 percent of the total observed decrease for very considerable modification. If this evidence is confirmed by similar infrared data at various intermediate temperatures, it will then be shown that 90 percent of the decrease in barriers to flow is realized when about 30 percent of the realizable weakening of the network is effected. Such a result would confirm the idea that the flow depends on a special distribution of the weakened bonds.

## 3. Conclusion

At some very high molten-glass temperature the units that flow may be very much smaller than vitrons and the process may more nearly resemble those which take place in liquids in general.^17^ However, it is here suggested that fissuring at the peripheries of vitrons is a basic phenomenon that provides space for cooperative maneuvers and governs a slip-channel process of vitreous flow in all silicate glasses at all moderately high temperatures where data have been taken.

Near annealing ranges, and in silica-rich glasses at higher temperature, flow is effected primarily by the breaking of tensed and weak Si–O bonds that are found chiefly in thin shell-like peripheral tissues surrounding the vitrons. In modifier-rich glasses, the shell-like tissues at very high temperatures may become channels populated with cations bonded to unshared oxygens and flow be effected primarily by the shearing of weakened cation-to-oxygen bonds. It is noticeable that in this structure range the activation energy for flow in alkaline earth silicates remains appreciably higher than that for the alkali silicates as would be expected if divalent cations, each bonded to two unshared oxygens, can hamper the relative movements of vitrons more than the monovalent cations.

For temperatures low in the annealing range it is suggested that viscous movements observable after holding are still between vitrons and surrounding network and that the increased activation energy is necessary for the cooperative movements. The immediately observable viscosity is attributed to volume relaxations, chiefly in the fissures, with the maximum relative travel between neighbors so small that the term flow is questionable and bending and yielding rather than breaking of the weakened bonds may be adequate. Both processes may operate in the intermediate regions of annealing ranges and the observed activation energies there may be sums.

An adequate understanding of vitreous flow in simple silicate glasses in definite terms of structure would involve data taken systematically on several types of glasses at numerous degrees of modification and at many temperatures including those near formation, flow region, softening region, annealing range, strain region, and perhaps lower temperatures.

This preliminary study utilizes ideas concerning fissuring, oxide-volume expansion, steric hindrance, Si-O-Si bond-angle contraction, and locally balanced stress systems (symmetry of smaller units versus vitron growth potential), which are largely developed from vitron theory and used in former applications thereof. These ideas have now been found qualitatively useful for tentative explanations of such salient trends in viscous flow, and in the activation energies therefor, as seem established by existing data.

## Footnotes

3 This vitron conception of the structure of silicate glasses is in accord with tentative conclusions reached by Warshaw from his electron microscopy of glass surfaces (J. Am. Ceram. Soc. 43, 4, 1960), and is well illustrated by his fig. 14 which is a schematic representation of the bulk glass showing the possible origin of the fractured surface which would yield the structure shown in the electron micrographs of his study.

4 Only dodecahedra that are radially decreased in diameter and elongated tangentially are usefully deformed for furthering growth of (spherical-type) vitrons in which all except a central cage must be so deformed as to touch and be integral with neighbor cages of the same shell, such as 12 around one.

5 H. T. Smyth (J. Am. Ceram. Soc. 42, 276, 1959) found that for silica-rich glasses, which have very low values of Poisson’s ratio, work must also be done in internal distortion of the network; whereas for some well-modified glasses, with largest values of Poisson’s ratio, it is necessary to assume that even more work is done in distorting the inclosed modifiers than in internal distortion of the network.

6 Very interesting possibilities are suggested by a comparison of this idea of fissuring with the concept of “fluctuating fissures” as proposed and illustrated by Jerzy Zarzycki (J. de Physique et le Radium 19, 13A, 1958) to accord with his analysis of radial distribution curves for molten salts.

7 This gives a slight flow corresponding to any stress, however small—a Newtonian flow. See H. T. Smyth, Glastechn. Berichte Sonderband 32K, III, 28, 1959.

8 See footnote, sec. 2.2.

9 This type of flow for glass is mentioned by W. A. Weyl, Rheology Vol. III, p. 338; Academic Press, Inc., New York. 1960.

10 A poise is the C.G.S. unit of absolute viscosity. In a liquid having unit viscosity a shearing force of 1 dyne will move a cm^2^ surface with a velocity of 1 cm per second relative to and parallel with a similar surface distant 1 cm transversely.

11 The broad regions (unfortunately sometimes called transformation points) where such bond-angle decrease in volume would be checked are prominent on the curves of volume versus temperature as plotted for well modified glasses. It should be noted, however, that no traces of such a region have been reported for pure silica glass. Moreover White [11] and Tomlinson et al. [22] have reported data from which one may infer that such configurational changes in volume may be absent also in binary silicates, both alkali and alkaline earth, having less than 10 to 15 percent nonsilica. Such a threshold for bond-angle sensitivity accords with the idea that some oxides, as such, can be inclosed in the cages and that only metal ions can deform the electron clouds and occasion bond-angle sensitivity. Existence of this threshold is evidenced by the continuous decrease in the partial molar volume of silica in silicate glasses with more than 12 or 13 percent R_2_O as reported by Callow [7]. See also lit. ref. 5, p. 16.

12 It will be noticed in figure 3 that the curve for *E_η_* of fused silica at and below annealing should be shifted to *lower* viscosities by 1 or 2 units in log *η* in order to be entirely consistent with the plotted values corresponding to the work of Jenckel and Schwittmann [24] and of Poole [25]. That the locus may be imprecise for fused silica will be realized from the dual results reported for log *η* by Volarovich and Leontieva [26] as plotted in figure 2. On the other hand, the rapid descent of *E_η_* for fused silica in figure 3 should correspond to a quasi-rigid condition such as is suggested by the other descending curves at somewhat *higher* viscosities on the right of the nominal annealing viscosity at log *η*=13.4. To the extent that these data are reliable, it is indicated that glasses very rich in silica have their maximum activation energies of flow at viscosities appreciably lower than those for well modified glasses; that is at temperatures relatively higher in their annealing ranges.

13 There are on record two series of unexplained experiments which indicate that the dominating expansional aspect of this relaxation during cooling is temperature sensitive down to about 200 °C in fused silica. The first series, by Douglas and Isard [37] consists of measured differences between the expansivities for specimens of quenched silica glass after heat treatments to equilibrium at several different temperatures from 990 ° to 1480 °C. At 200 ° to 1000 °C the expansivities are numerically greater for the specimens which had higher-temperature heat treatments (and yielded greater values of densities). Below 175 ° or 200 °C the differences are negligible. The second series, the compressibilities of silica glass at various temperatures from 0 to 400 °C and for 5 different pressure steps (F. Birch and R. B. Dow, Bull, Geol. Soc. Amer. 47, 1235, 1936) shows not only that compressibilities become higher at increased pressures [1, p. 149], and that they decrease with increasing temperature, but that the decrease with temperature is most rapid near 175 ° to 200 °C. (See lit. ref. 1, p. 149; also C. L. Babcock, Stephen W. Barber, and Kasimir Fajans, Ind. and Eng. Chem. 46, 161, 1954.)

14 See table 1 and fig. 13 of paper cited in lit. ref. 2. Although table 1 was proposed for a silica glass structure consisting predominantly of vitrons, the principal of equidistribution of modifier oxide throughout the silica permits the table to to apply also to the vitron clusters immersed in denser network, to the residual denser network itself, and to the whole glass.

15 The writer has suggested that no bridges are broken until modifier content exceeds “oxide saturation.” See Section 2.1 of paper cited in lit. ref. (2). It now seems necessary to modify this by adding, “except possibly in the interconnective or channel fissures, probably a small proportion of total volumes.”

16 It has been noted by C. R. Kurkjian and R. W. Douglas (Physics and Chemistry of Glasses 1, 19, 1960) that few measurements on dilute solutions of alkali oxides in silica have been made because of the high temperatures involved, but their results with dilute solutions for Na_2_O-GeO_2_ glasses lead them to conclude that “only of the order two percent alkali is required to produce a complete change in the flow mechanism at high temperatures.”

17 This may be the explanation of the decrease in slope of the curve of log *η* versus 1/*T* at very high temperatures where such curves tend to converge and where it seems possible that vitrons are formed from discrete tetrahedra.
